# Defining recovery from anorexia nervosa: a Delphi study to explore practitioners' views

**DOI:** 10.1186/2050-2974-1-S1-O41

**Published:** 2013-11-14

**Authors:** Lisa Dawson, Paul Rhodes, Stephen Touyz

**Affiliations:** 1 The University of Sydney, Australia

## 

There is no consensus as how best to define and measure recovery from anorexia nervosa (AN). Definitions of recovery vary vastly between studies, making them difficult to compare. This has been identified as a major barrier in the field. The aim of the current study was to explore how best to define and measure recovery according to expert researchers and clinicians. This research used the Delphi technique, a method used to gain consensus on an issue. Twenty leading international experts in the field completed three rounds of online questionnaires. This was a multi-stage process with each round building on the previous round until consensus was achieved amongst the panel. Preliminary findings have revealed that experts in the field define recovery from AN as more than the restoration of weight and absence of eating disordered behaviours. Participants suggested that recovery also includes experiencing normal levels of body dissatisfaction and improved quality of life and social functioning. Full findings are reported and the research and clinical implications for establishing a consensus definition in the field are discussed.

This abstract was presented in the **Anorexia Nervosa – Characteristics and Treatment** stream of the 2013 ANZAED Conference.

